# Survival Outcomes in Hepatocellular Carcinoma: Experience from a Multidisciplinary Committee in Ecuador

**DOI:** 10.3390/life15101565

**Published:** 2025-10-08

**Authors:** Enrique Carrera, Jaysoom Abarca, Johana Acuña, Mercedes Almagro, David Armas, Cinthya Borja, Wendy Calderón, Diana Chamorro, Daniel Garzon, Melina Gonzalez, Andrea Moreno, Mónica Proaño, Darwin Quevedo, Maritza Quishpe, Juan Fernando Salazar, Fabian Tulcanazo, Cecilia Trujillo, Gabriela Velalcazar

**Affiliations:** 1 Department of Gastroenterology and Hepatology, Hospital Especialidades Eugenio Espejo, Quito EC 170136, Ecuadormercedesalmagror@gmail.com (M.A.);; 2 School of Medicine, Universidad San Franciso de Quito, Pampite and Diego Robles, Quito EC 170901, Ecuador; dgarzonc@usfq.edu.ec; 3 School of Medicine, Pontificia Universidad Católica del Ecuador, Av. 12 de Octubre 1076 and Vicente Ramón Roca, Quito EC 170525, Ecuador; 4 Post-Graduation Program in Hepatology, Universidade Federal de Ciências da Saúde de Porto Alegre, Sarmiento Leite, 245, Porto Alegre 90050-170, Brazil; joha.acuna16@gmail.com

**Keywords:** liver, hepatocellular carcinoma, survival, committee

## Abstract

Hepatic cancer is a world health concern due to its high lethality. The main risk factor worldwide is having hepatic cirrhosis. The etiology of hepatic cirrhosis has changed in recent years, with metabolic-associated steatotic liver disease (MASLD) becoming the leading cause, displacing hepatitis C and B viruses and alcoholic liver disease. It is of the utmost importance to develop screening programs in at-risk populations for early detection. The survival rate of HCC, as determined by a group of specialists or an interdisciplinary committee, is a challenge we have taken on in a public health hospital in Ecuador. This retrospective study identified 71 patients diagnosed with hepatocellular carcinoma, mostly middle-aged men with a history of liver cirrhosis. No significant association was found between the presence of cirrhosis, laboratory abnormalities, and survival. However, the identification by imaging vascular invasion and extrahepatic extension were associated. This study highlights that patients with liver lesions identified through HCC screening have a higher survival rate over a one-year follow-up period.

## 1. Introduction

Hepatocellular carcinoma (HCC) is the most common primary liver cancer, characterized by its high mortality rate. It is the sixth most common cancer worldwide and the third leading cause of cancer death globally [1].

In Ecuador, the incidence rate published in the national tumor registry is 4.6 cases per 100,000 inhabitants, which is lower than the rest of South America and much higher than the rates in East Asia and North Africa. Regarding mortality, Ecuador has a rate of 4.4 cases per 100,000 inhabitants for both sexes [2]. This lower incidence of HCC in Ecuador is unfortunately underestimated because there is no national or regional policy for the management of liver cancer; all records go into a large database coordinated by INEC (National Institute of Statistics and Census); however, many sub-records are kept in third-level hospitals, for this, the overall statistics are not accurate. Due to this inconvenience, our liver and biliary tract tumor committee upholds a more real and measurable quantification of cases with diagnosis of liver cancer since 2018, when it was created.

Most cases arise in developing countries, especially in Asia and Africa, where high rates of chronic hepatitis B and C infections, as well as exposure to aflatoxins, contribute significantly to the burden of this disease. In developed countries, risk factors such as alcohol consumption and obesity, which lead to metabolically impaired steatosis liver disease (MASLD), contribute to the increased incidence of HCC [3].

In the Americas, HCC represents a significant challenge for both public and private health systems. Although incidence rates are comparatively lower than in other regions, such as Asia and Africa, HCC still represents a major issue in public health. The prevalence of risk factors varies among countries in the region: some have higher rates of viral hepatitis and alcohol-related liver disease, while in recent years, MASLD has become the main risk factor for chronic liver disease and its complications [3,4].

Various treatments have been proposed for the management of HCH, based primarily on the patient’s characteristics, focusing on whether or not they have cirrhosis, portal hypertension, their functional stage determined by the Child–Pugh scale, one or more lesions, and if they have vascular involvement. The staging system of The Barcelona Liver Cancer Clinic (BCLC) is the most common and popular in America, it is considered a great guide that should be adapted to the needs and realities of each country, which leads us to a better management of curative and palliative therapies [5]. The objective of this study is to evaluate the factors related to the survival of patients with hepatocellular carcinoma evaluated by the interdisciplinary committee formed in a tertiary hospital in Quito, Ecuador. The findings will provide a valuable contribution to the global body of literature on liver cancer.

## 2. Methods

This is a retrospective cohort study of patients seen in the outpatient clinic and/or hospitalized at a national public health hospital of significant importance (Eugenio Espejo Hospital) in Quito, Ecuador, between 2016 and 2023. All cases were evaluated by a liver and biliary tract tumor committee.

This study included patients with hepatocellular carcinoma (HCC) who were diagnosed according to the guidelines of the American Association for the Study of Liver Diseases (AASLD) [6] and the European Association for the Study of the Liver (EASL) [7]. Patients with cirrhosis, regardless of the cause, as well as those who developed HCC without underlying chronic liver disease, were also included. In patients with cirrhosis and suspected HCC, contrast studies such as magnetic resonance imaging or computed tomography were used and LI-RADS (Liver Imaging Reporting Data System) criteria were applied. For patients without cirrhosis, the diagnosis was established through biopsy. All patients were evaluated through a multidisciplinary approach by a dedicated committee.

Pregnant patients and minors were excluded from the study. We recorded demographic data, the cause of underlying liver disease, comorbidities, family history of HCC, method of diagnosis, treatment, clinical progression, and one-, two-, and three-year survival rates. For imaging, we focused on variables such as tumor count, the diameter of the largest tumor, vascular invasion, and extrahepatic lesions.

Data were extracted from medical records by the hospital’s statistics department and, to preserve anonymity, all analyses were performed using a coded database. Descriptive and inferential statistics were computed using R Pack and Microsoft Excel. The patients were part of the ESCALON Project, a collaborative network between Latin America and Europe focused on hepatobiliary cancer, and provided written consent for their participation in this and related studies.

## 3. Results

Seventy-one patients were included in this study for demographic and laboratory variables, and seventy patients were considered for the survival analysis. The sample consisted predominantly of males, with a male-to-female ratio of 1.15, and the median age of patients was 67.85 years, ranging from 21 to 89 years. Table 1 presents information on ethnic groups, age, level of education, habits, and comorbidities. Finally, the most affected ethnic group was mestizos or Hispanics in 70 cases (98.6%).

In patients with cirrhosis, most cases were classified as Child–Pugh A and B, with a smaller proportion classified as Child–Pugh C. Additionally, almost two-thirds of the patients were classified as BCLC stages A or B. All stages were considered for the study. Table 2 shows the distribution of patients according to Child–Pugh and BCLC classifications.

Patients included in this study were diagnosed with HCC after clinical manifestations in 81.7% (n = 51) of cases and by surveillance in 18.3% (n = 13) of cases. Surveillance protocol follows the standard recommendation of international societies of use of ultrasound in addition to alpha-fetoprotein every six months.

No significant association was found between the presence of cirrhosis or laboratory test results and the survival rate at the end of study. However, serum albumin values were higher in patients that died at one year than in those who survived (3.6 ± 0.947 vs. 3.14 ± 00.581, *p* = 0.015). Table 3 describes laboratory test results according to the presence of cirrhosis.

Treatments received by the patients are shown in Table 4; these include palliative, pharmacological, or surgical procedures.

In this study, the mean survival time since diagnosis was 13.69 months (410.87 days) with a Std. Deviation of 483.15 (minimum 1–maximum 2065) (CI 95%); with 22 out of the 70 patients still alive by the end of the data collection and 2.82% of patients alive at 5 years.

**Figure 1:**
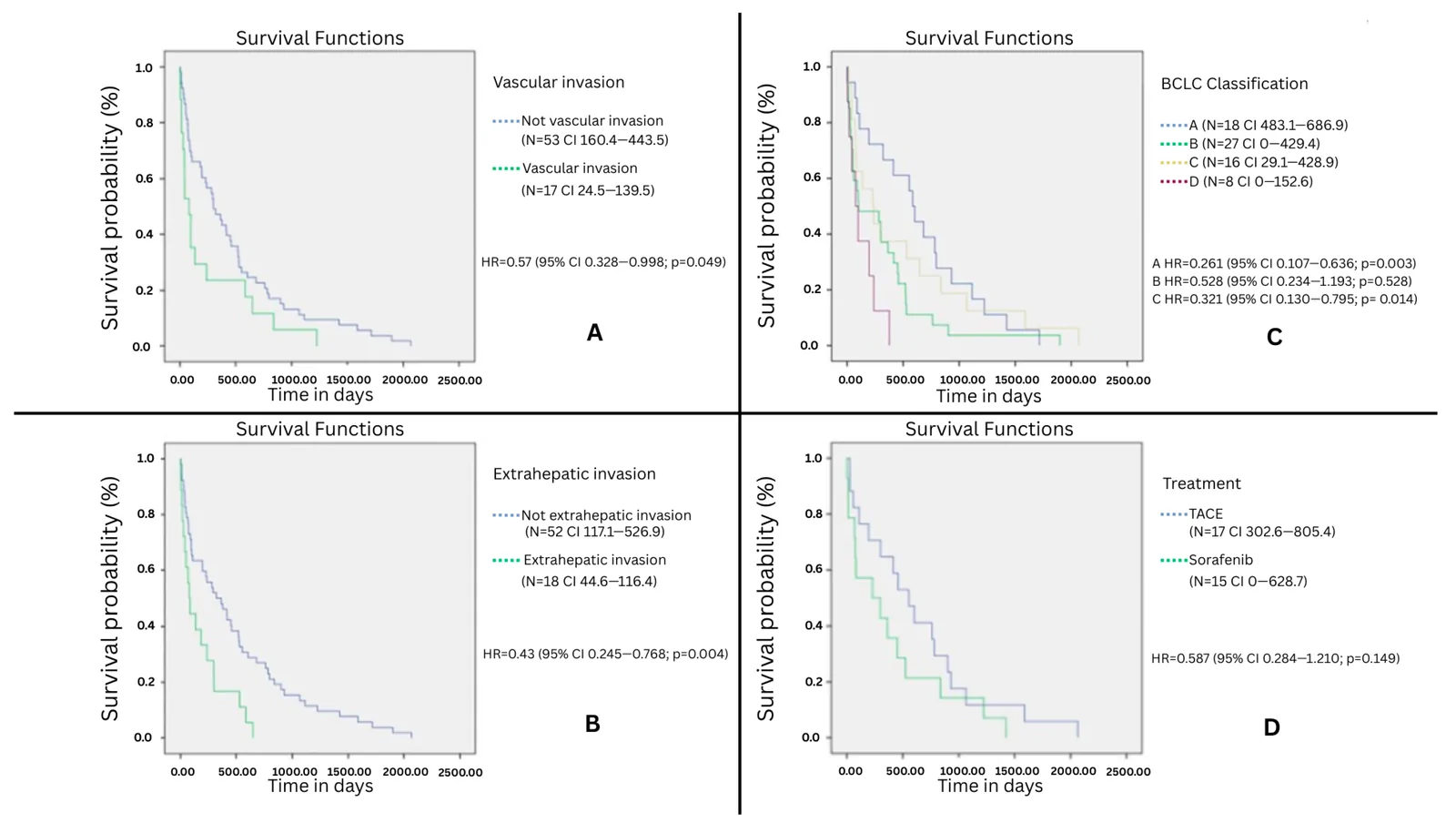
Kaplan–Meyer test survival plot, *x*: time in days, and *y*: percentage of patients alive in function of time. (**A**) Survival according to the presence of intravascular invasion, (**B**) survival according to the presence of extravascular spread, (**C**) survival according to the BCLC classification, (**D**) comparison between TACE (n = 17) and sorafenib (n = 15).

Kaplan–Meyer shows that vascular invasion was significantly related to survival time in days (X^2^ = 3.98, dL = 1, *p* = 0.046), as well as the extra-hepatic spread (X^2^ = 8.7, dL = 1, *p* = 0.003). No association was found between the Child–Pugh classification and survival at the end of 5 years; there is an association only at the end of the one year and there was also a significant association with BCLC categories (X^2^ = 11.66, dL = 3, *p* = 0.009). Comparing survival and treatment, no significant correlations were found (X^2^ = 1.754, dL = 1, *p* = 0.185). Nevertheless, a better survival rate at 500 days was observed with TACE (Figure 1). Comparing cases identified by screening with those identified by clinical manifestations, patients identified while on screening had better survival rates (Mantel–Cox log rank X^2^ = 7.394, dL = 1, *p* = 0.007).

A binary logistic regression was performed by steps, showing that independent variables (Age, stage Child–Pugh/BCLC scale, cirrhosis and treatment) over the probability of occurrence in four steps of dependent variable (X^2^ = 33.493, df = 11 *p* ≤ 0.001) explain the 0.26% of change over the dependent variable (R2 Nagelkerke = 0.51). All the effects of each variable are displayed in the Supplementary Table S1; Child–Pugh category and treatment are the two variables that were kept after the four steps of the model, RFA treatment present an odds ratio of 23.6, associated with surviving the first year. Child–Pugh category presents an accumulated significance; however, by categories, only Child–Pugh C is related with an odds ratio 0.086, suggesting association with death before the first year, but its significance is only in the limit (*p* = 0.051).

We performed a Cox proportional-hazards model adjusting of BCLC stage (X^2^ = 11.989, df = 4, *p* = 0.017), where presence of cirrhosis presents a non-statistically significant value (B/SE = −0.177, 0.292, Wald = 0.366, *p* = 00.545, CI 0.472–1.486); this confirms that cirrhosis is not associated with survival.

## 4. Discussion

Hepatocellular carcinoma (HCC) is the most common primary liver cancer, with a high regional and global impact. This study, the first of its kind to be conducted in Ecuador’s public health system with the participation of a multidisciplinary committee, examined the demographic characteristics and survival factors of patients with HCC.

Similarly to what was found in the present study, almost 80% of HCC cases develop in the context of a cirrhotic liver, which, by having a cellular alteration associated with fibrosis, creates a chronic inflammatory state, which leads to DNA alterations and abnormal cell proliferation; this is independent of the underlying etiology of chronic liver disease, with an estimated annual incidence of 2% to 4% [8,9].

In our study, approximately 21% of hepatocellular carcinoma (HCC) patients were non-cirrhotic. The majority of these patients presented diabetes and metabolic dysfunction-associated steatotic liver disease (MASLD). This finding is not surprising, as surveillance is well-established for cirrhotic patients of any etiology, as well as for those with HBV and HCV infections, but not for the other risk factors for chronic liver disease. A similar incidence has been previously reported. A large retrospective study of 1400 HCC patients over a 10-year period identified at least 17% of patients as non-cirrhotic. Recent reports suggest that this population is growing [10,11]. Non-cirrhotic HCC shows a distinct age distribution with a double peak, one peak occurring in the second decade and a second peak in the seventh decade of life. Furthermore, the fibrolamellar subtype is the most common in this population [12]. Hepatocellular carcinoma (HCC) subtypes were not included in our results. This was because histological data were not consistently available for all patients, and the diagnosis was based on clinical and radiological evidence.

In this retrospective study, 71 cases of HCC were identified at a national public health hospital of significant importance. The male-to-female ratio was 1.15, with the average age of 67 years. This male predisposition is a tendency that has already been demonstrated in epidemiological and survival studies, with some studies reporting ratios as high as 2 to 1 [13,14]. At 5 years, HCC has an overall 5-year survival rate of 18%, with reported geographic variations. In Europe, the United Kingdom has one of the highest 5-year survival rates, at 12.1%. In the United States, this rate reaches 8.1% over the same period [14,15]. In Latin America, Argentina reports a 36% survival rate, while Colombia and Chile report average survival times of 90.5 and 6.3 months, respectively.

Although a higher proportion of deaths occurred in patients with cirrhosis, we found no significant association between cirrhosis and 3-year survival. This lack of statistical significance may be due to the small sample size of non-cirrhotic patients in our study.

Among the laboratory tests, levels of albumin, lymphocytes, INR, and total bilirubin were associated with first-year survival. In other studies, various tumor parameters, such as size or vascular invasion, were associated with albumin levels below 30.5 mg/dL, suggesting that lower albumin levels were correlated with more aggressive tumors, thereby indicating a poorer prognosis [16,17]. In this study, alpha-fetoprotein (AFP) levels were not associated with mortality; however, there was a statistically significant difference between the survival and death groups in the first, second, and third years (Supplementary Table S2). This finding aligns with previous reports indicating that levels exceeding 1000 ng/mL are associated with a poorer prognosis and higher recurrence rates [18].

Regarding staging, the majority of our patients (63.4%) were classified as stage A or B, according to the BCLC staging system, making them candidates for curative treatment. This staging significantly correlated with survival time. However, only 7% of patients underwent curative treatments, including liver transplantation or surgical resection. Notably, a high percentage (12.7%) did not receive any treatment, and approximately one-third were diagnosed at an advanced stage, requiring only palliative care.

We did not include an analysis of the factors that could explain delays in accessing treatment. A large retrospective study conducted in the late 1990s and early 2000s showed that low socioeconomic status was associated with reduced survival rates. This study also found that this trend has been increasing over time [19].

A key variable to consider is that our hospital is public and of national reference that offers specialized care at the third level; consequently, the vast majority of patients in this study were referred from various healthcare systems across Ecuador, taking into account that the administrative cooperation process between healthcare levels leads to significant diagnostic delays. Primary care facilities lack the resources for a definitive liver tumor diagnosis and must refer patients to an intermediate level for imaging and laboratory tests. Without having an anatomopathological study of a biopsy, there is a significant time delay for an efficient diagnosis of cancer. This issue is likely prevalent across Latin America, revealing a clear disparity in diagnosis and treatment of HCC. Argentina and Brazil found that 25% and 86% of patients were unaware of their chronic liver disease before HCC diagnosis. It shows that the first step to improve our results in HCC management is to identify the susceptible population who needs to be included in surveillance programs [20]. However, a multicenter cohort study in the United States shows that just 14% of patients received semiannual surveillance and 22.3% received annual surveillance [21]. Therefore, perhaps globally, we need to better identify patients and standardize not only screening methods but also their access. An analysis of the social determinants of health indicates that patients from low-income areas exhibit both a higher incidence of HCC and lower survival rates, compared to the general population. This disparity is partially attributable to limited access to healthcare, as previously mentioned, as well as several contributing factors such as inadequate health education, failure to adhere to liver pathology screening programs, and significant delays in obtaining six-month abdominal ultrasound appointments as per a scarcity of hepatology specialists at the second level of care. Ultimately, these issues have a negative impact on the overall management of liver cancer.

Access to healthcare systems or health insurance has a significant impact on HCC outcomes. This analysis showed a median overall survival of 34 months for patients with private health insurance, compared to 9 months for those without it [22].

Among the patients who received treatment, although a significant correlation was not observed between treatment type and survival, those who underwent transarterial chemoembolization (TACE) showed higher survival rates. The absence of statistical significance could also be attributed to the small sample size. Local-regional treatment is available worldwide and includes transarterial chemotherapy and radioembolization (TARE). Even though TACE is the most widely used treatment, TARE appears to be a promising therapy with a positive impact on overall survival in patients with intermediate-stage HCC and also in patients with advanced-stage HCC, but with limited extension to survival [23,24]. It is worth mentioning that the use of chemotherapeutic agents such as lenvatinib or atezolizumab plus bevacizumab has shown a positive effect on the survival of patients undergoing conversion therapy, especially those with BCLC stage B [25]. Furthermore, no stage reduction was reported among our patients.

Patients in whom liver lesions were identified and diagnosed with HCC following active surveillance protocols had better survival rates than those whose diagnosis was prompted by clinical manifestations (Supplementary Table S1). This finding is consistent with the ones presented by a meta-analysis of 47 studies, where 50.8% of patients undergoing surveillance survived for 3 years, compared to 28.2% of patients who were not monitored [26]. Unfortunately, the lack of a national HCC surveillance guide prevents different health systems from identifying which patients need to be screened for this tumor and how often.

This outcome also reinforces the importance of a multidisciplinary approach to patients with liver injury. Such an approach, which extends beyond a mere protocol, facilitates earlier and more accurate diagnoses, leading to a longer life expectancy [27]. The benefits of this approach are evident even in patients with poor liver function, elevated alpha-fetoprotein (AFP) levels, and advanced tumor stages [28].

The involvement of a multidisciplinary committee has been associated with improved outcomes in other types of gastrointestinal cancer, including lower recurrence rates and enhanced disease-free survival [29]. Thirty years ago, the Calman–Hein report recommended restructuring cancer services to include multidisciplinary management and ensure equitable access to care for the cancer population [30].

## 5. Conclusions

Hepatocellular carcinoma (HCC) is one of the leading causes of cancer-related death worldwide. The epidemiology of chronic liver disease risk factors is changing, particularly due to the growing obesity epidemic and the prevalence of metabolically associated steatosis liver disease (MASLD). This study found that men, with an average age of approximately 67 years, had the highest rates of HCC and the diagnosis was more frequently made after patients presented with symptoms rather than through surveillance protocols for cirrhotic patients. Our median overall survival was 13.86 months. We observed better outcomes in patients who received TACE within the first 500 days of follow-up. The most important limitation of our study is the sample’s size. The sample included just 71 patients, despite the study being conducted in a national reference center. This small sample limits the generalization of the results. Further studies are needed to validate these findings and explore other contributing factors, such as treatment delay.

## Figures and Tables

**Table 1:** Demographic characteristics of patients with hepatocellular carcinoma.

	Male	Female
Sex	n = 38, 53.3%	n = 33, 46.5%
Age in years (median minimum/maximum)	68.13 (44, 89)	67.52 (21, 63)
Education Level		
Analphabet	n = 1, 2.63%	n = 3, 7.89%
Elementary	n = 25, 65.79%	n = 23, 60.53%
Secondary	n = 10, 26.32%	n = 4, 10.53%
University	n = 2, 5.26%	n = 3, 7.89%
Comorbidities		
Diabetes	n = 10, 26.32%	n = 10, 26.32%
Hypertension	n = 14, 36.84%	n = 12, 31.58%
Renal disease	n = 3, 7.89%	n = 1, 2.63%
Habits		
Alcohol	n = 6, 15.79%	n = 0
Tobacco	n = 3, 7.89%	n = 0
Body Mass Index (median minimum/maximum)	n = 34 (27.13, 20–38)	n = 26 (29, 20–43)
Ethnic group		
Mestizo	n = 70, 98.6%	
Indigenous	n = 1, 1.4%	

Alcohol was defined as the presence or not of historical alcohol consumption, limited data does not allow us to estimate grams per day. Tobacco consumption was defined as the presence or not of historical consumption. Ethnic groups were self-identified.

**Table 2:** Classification of patients by Child–Pugh and BCLC Staging.

Child–Pugh	A	n = 21, 29.6%
	B	n = 27, 38%
	C	n = 8, 11.3%
	no cirrhosis	n = 15, 21.21%
BCLC	A	n = 18, 25.4%
	B	n = 27, 38%
	C	n = 17, 23.9%
	D	n = 8, 11.3%

**Table 3:** Laboratory test results according to the presence of cirrhosis.

Laboratory Tests	No Cirrhosis (Median Minimum/Maximum)	Cirrhosis (Median Minimum/Maximum)	Statistical Test
AST (U/L)	78.4 (29–172)	115.04 (28–909)	*p* = 0.453
ALT (U/L)	43.27 (13–109)	69.69 (12–396)	*p* = 0.134
Total Bilirubin (mg/dL)	1.83 (0.42–7.25)	2.82 (0.57–13.9)	*p* = 0.06
INR	1.1 (1.02–1.2)	1.27 (1–1.86)	*p* < 0.001 *
ALP (U/L)	318.67 (99–1370)	287.75 (106–943)	*p* = 0.563
Hemoglobin (g/dL)	13.8 (7–18.8)	16.35 (6.2–168)	*p* = 0.582
Platelets (×10^3^/μL)	324.8 (118–710)	148 (47–688)	*p* < 0.001 *
Leucocytes (×10^3^/μL)	8.6 (4.9–15.18)	7.6 (1.7–55.06)	*p* = 0.620
% Neutrophils	65.85 (42.7–85)	60.17 (2.24–81.4)	*p* = 0.234
% Lymphocytes	20.67 (5.7–45.4)	23.88 (9–44)	*p* = 0.256
Creatinine (mg/dL)	1.34 (0.5–9)	1.42 (0.37–27)	*p* = 0.751
NA (mEq/L)	135.47 (122–147)	135.33 (128–144)	*p* = 0.749
AFP	5624.86 (2.06–50,000)	7398 (1.3–50,000)	*p* = 0.260
Albumin (g/dL)	3.6 (2–5)	3.24 (0–5)	*p* = 0.68

Leucocytes, % Neutrophils, NA, Albumin present a normal distribution according to Kolmogorov–Smirnov (in these variables we used Student’s *t*-test), all other laboratory variables were analyzed using Mann–Whitney U test. AFP has 50,000 as upper level because of laboratory limit of detection. * Indicates significant statistic correlation. ALT: alanine aminotransferase; AST: aspartate aminotransferase; INR: International normalized ratio; ALP: alkaline phosphatase; NA: sodium; AFP: alpha-fetoprotein.

**Table 4:** Treatments offered for hepatocellular carcinoma.

	Number/Percentage
No treatment	n = 9, 12.7%
TX	n = 2, 2.8%
Resection	n = 3, 4.2%
TACE	n = 17, 23.9%
RFA	n = 1, 1.4%
Sorafenib	n = 15, 21.1%
Gemcitabine-oxaliplatin	n = 1, 1.4%
Palliative	n = 22, 31%

## Data Availability

The original contributions presented in this study are included in the article/Supplementary Material. Further inquiries can be directed to the corresponding author.

## Supplementary Materials

The following supporting information can be downloaded at: https://www.mdpi.com/article/10.3390/life15101565/s1, Figure S1: Kaplan-Meier survival analysis by treatment; Table S1: Annual survival and diagnosis by symptomatology; Table S2: Laboratory and survival association; Table S3: Multivariate logistic regression analysis of factors associated with first-year survival in patients with hepatocellular carcinoma.
