# Supplementary material for: Survival Outcomes in Hepatocellular Carcinoma: Experience from a Multidisciplinary Committee in Ecuador
Source: Life (Basel). 2025 Oct 8;15(10):1565. doi: 10.3390/life15101565 (PMC12565213; doi:10.3390/life15101565)
Supplement: Supplementary file 1 [file life-15-01565-s001.zip › life-3845976-supplementary.pdf]

Supplementary Table S1

	year 1	year 2	year 3	year 4	year 5
Patients number that survive	28 (39.44%)	15 (21.13%)	7 (9.86%)	4 (5.63%)	2 (2.82%)
Child-Pugh	p=0.013*	p=0.305	p=0.608	p=0.880	p=0.351
Surveillance / symptomatology diagnosis	p=0.259	p=0.016*	p=0.006	p=0.096	p=0.003

*Survive more in the group of patients identified by sintomatology
Pearson Chi-Square

Supplementary Table S2

**Laboratory and survival association**	**Survival**				
**Laboratory test**	year 1 (n=28)	year 2 (n=15)	year 3 (n=7)	year 4 (n=4)	year 5 (n=2)
Total Billirrubine	p=0.016*	p=0.056	p= 0.411	p= 0.990	p= 0.199
INR	p=0.038*	p=0.076	p= 0.984	p= 0.183	p= 0.438
Linfocites	p=0.017*	p=0.016*	p= 0.769	p= 0.617	p= 0.268
AST	p=0.122	p=0.05*	p= 0.762	p= 0.817	p=0.902
ALT	p=0.135	p=0.044*	p= 0.945	p= 0.817	p = 0.497
AFP	p= 0.011*	p=0.01*	p=0.021*	p=0.061	p=0.451
Albumine [1]	p=0.015*	p=0.110	p=0.410	p= 0.756	p= 0.553

Presence of statistical significant difference, the test used was Mann-Withney U, only Alfa feto protein present difference until the third year, after this point the group that survive is inferior of 5 years. [1] Test used was T student test.

Supplementary Table S3

		B	S.E.	Sig.	Odds Ratio	95% C.I.for EXP(B)	
						Lower	Upper
Step 1[a]	Age	-0.024	0.034	0.483	0.976	0.913	1.044
	Treatment			0.317			
	Not treatment	-0.483	1.618	0.765	0.617	0.026	14.718
	TX	23.789	24,632.562	0.999	2.145E+10	0.000	
	TACE	2.000	2.181	0.359	7.389	0.103	531.448
	RFA	2.887	1.623	0.075	17.944	0.746	431.851
	Sorafenib	21.322	40,192.970	1.000	1.820E+09	0.000	
	Gemcitabine/oxiplatine	0.159	1.468	0.914	1.173	0.066	20.852

	Paliative	-18.310	40,192.970	1.000	0.000	0.000	
	TACE paliative	0.366	1.937	0.850	1.442	0.032	64.181
	Child-pug			0.044			
	Child-pug A	-22.139	40,192.972	1.000	0.000	0.000	
	Child-pug B	0.734	1.390	0.597	2.084	0.137	31.758
	Child-pug C	-2.276	1.343	0.090	0.103	0.007	1.428
	Chirrosis	21.841	40,192.972	1.000	3.058E+09	0.000	
	BCLC scale			0.714			
	BCLC scale A	0.955	1.698	0.574	2.599	0.093	72.419
	BCLC scale B	-0.105	1.713	0.951	0.900	0.031	25.867
	BCLC scale C	0.619	1.722	0.719	1.856	0.064	54.266
	Constant	0.197	4.139	0.962	1.218		
Step 2[a]	Age	-0.021	0.031	0.503	0.979	0.922	1.041
	Treatment			0.242			
	Not treatment	-0.089	1.557	0.955	0.915	0.043	19.362
	TX	23.640	25,813.806	0.999	1.849E+10	0.000	
	TACE	2.711	2.021	0.180	15.047	0.287	789.600
	RFA	2.983	1.587	0.060	19.751	0.880	443.151
	Sorafenib	21.853	40,192.970	1.000	3.095E+09	0.000	
	Gemcitabine/oxiplatine	0.368	1.455	0.800	1.445	0.083	25.009
	Paliative	-17.881	40,192.970	1.000	0.000	0.000	
	TACE paliative	0.223	1.650	0.892	1.250	0.049	31.702
	Child-pug			0.024			
	Child-pug A	-21.709	40,192.968	1.000	0.000	0.000	
	Child-pug B	0.631	1.252	0.614	1.879	0.161	21.869
	Child-pug C	-2.540	1.264	0.045	0.079	0.007	0.940
	Chirrosis	21.153	40,192.968	1.000	1.537E+09	0.000	
	Constant	0.464	2.843	0.870	1.590		
Step 3[a]	Treatment			0.204			
	Not treatment	-0.044	1.534	0.977	.957	0.047	19.368
	TX	24.044	26,836.619	0.999	2.769E+10	0.000	
	TACE	2.737	2.009	0.173	15.440	0.301	792.310
	RFA	3.077	1.563	0.049	21.692	1.013	464.272
	Sorafenib	21.567	40,192.970	1.000	2.325E+09	0.000	
	Gemcitabine/oxiplatine	0.382	1.431	0.789	1.465	0.089	24.209
	Paliative	-17.680	40,192.970	1.000	0.000	0.000	
	TACE paliative	0.190	1.624	0.907	1.210	0.050	29.151
	Child-pug			0.027			
	Child-pug A	-21.646	40,192.966	1.000	0.000	0.000	
	Child-pug B	0.690	1.239	0.578	1.994	0.176	22.627
	Child-pug C	-2.469	1.263	0.051	0.085	0.007	1.007
	Chirrosis	21.185	40,192.966	1.000	1.587E+09	0.000	
	Constant	-1.054	1.720	0.540	0.348		
Step 4[a]	Treatment			0.214			
	Not treatment	-0.041	1.544	0.979	0.960	0.047	19.810
	TX	24.141	26,865.132	0.999	3.049E+10	0.000	
	TACE	2.817	2.024	0.164	16.729	0.316	884.465
	RFA*	3.162	1.576	0.045	23.608	1.076	517.814

Sorafenib	21.534	40,192.970	1.000	2.249E+09	0.000	
Gemcitabine/oxiplatine	0.598	1.420	0.673	1.819	0.112	29.426
Paliative	-17.588	40,192.970	1.000	0.000	0.000	
TACE paliative	0.280	1.630	0.863	1.323	0.054	32.304
Child-pug*			0.020			
Child-pug A	-0.467	1.213	0.700	0.627	0.058	6.759
Child-pug B	0.831	1.231	0.499	2.297	0.206	25.632
Child-pug C	-2.453	1.259	0.051	0.086	0.007	1.015
Constant	-1.162	1.723	0.500	0.313		

RFA treatment present a odds ratio of 23.6 probabilities of survive the first year.

Supplementary Figure S1

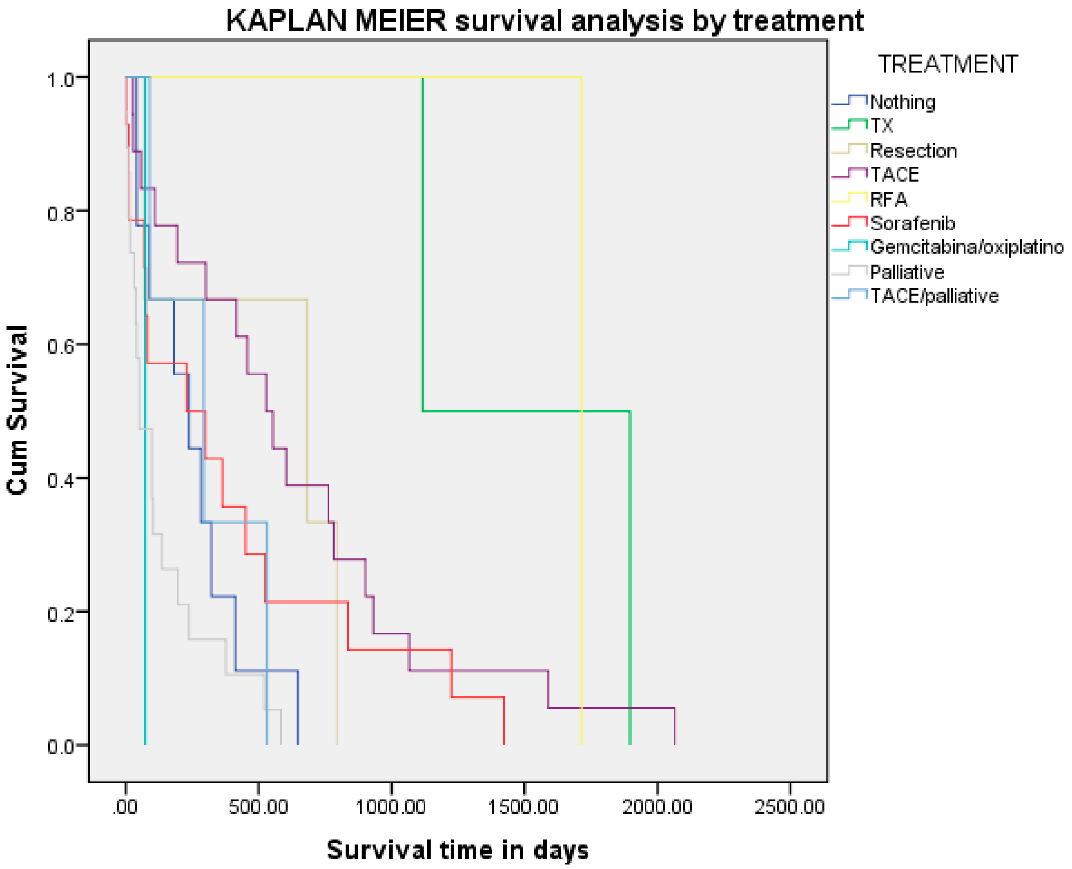

Kaplan-Meier survival analysis by treatment shows statistical differences between treatment applied, (Long Rank (Mantel-cox) Chi-Square = 29.801, df = 8 p<0.001), with the following confidence intervals: nothing (74.4-401.6), TX (only one case), Resection (0-1659.8), TACE (328.4-727.6), RFA (only one case), Sorafenib (0-628.7), Gemcitabina + oxiplatine (only one case), Palliative (0-110.7), TACE+palliative (0-620.5). Overall (117.1-358.9).
